# Deep Learning Model for Volume Measurement of the Remnant Pancreas After Pancreaticoduodenectomy and Distal Pancreatectomy

**DOI:** 10.3390/diagnostics15222834

**Published:** 2025-11-08

**Authors:** Young Jae Kim, Juhui Lee, Yeon-Ho Park, Jaehun Yang, Doojin Kim, Kwang Gi Kim, Doo-Ho Lee

**Affiliations:** 1 Gachon Biomedical & Convergence Institute, Gachon University Gil Medical Center, Incheon 21565, Republic of Korea; kimyj@gilhospital.com; 2 Medical Devices R & D Center, Gachon University Gil Medical Center, Incheon 21565, Republic of Korea; juhui05134@gmail.com; 3 Department of Surgery, Gachon University Gil Medical Center, Gachon University College of Medicine, Incheon 21565, Republic of Korea; pyh@gilhospital.com (Y.-H.P.); gsyangjh@gilhospital.com (J.Y.); drkdj@gilhospital.com (D.K.); 4 Department of Biomedical Engineering, Gachon University Gil Medical Center, Gachon University College of Medicine, Incheon 21565, Republic of Korea

**Keywords:** pancreas, volumetry, deep learning, computed tomography, segmentation

## Abstract

**Background/Objectives:** Accurate volumetry of the remnant pancreas after pancreatectomy is crucial for assessing postoperative endocrine and exocrine function but remains challenging due to anatomical variability and complex postoperative morphology. This study aimed to develop and validate a deep learning (DL)-based model for automatic segmentation and volumetry of the remnant pancreas using abdominal CT images. **Methods:** A total of 1067 CT scans from 341 patients who underwent pancreaticoduodenectomy and 512 scans from 184 patients who underwent distal pancreatectomy were analyzed. Ground truth masks were manually delineated and verified through multi-expert consensus. Six 3D segmentation models were trained and compared, including four convolution-based U-Net variants (basic, dense, residual, and residual dense) and two transformer-based models (Trans U-Net and Swin U-Net). Model performance was evaluated using five-fold cross-validation with sensitivity, specificity, precision, accuracy, and Dice similarity coefficient. **Results:** The Residual Dense U-Net achieved the best performance among convolutional models, with dice similarity coefficient (DSC) values of 0.7655 ± 0.0052 for pancreaticoduodenectomy and 0.8086 ± 0.0091 for distal pancreatectomy. Transformer-based models showed slightly higher DSCs (Swin U-Net: 0.7787 ± 0.0062 and 0.8132 ± 0.0101), with statistically significant but numerically small improvements (*p* < 0.01). **Conclusions:** The proposed DL-based approach enables accurate and reproducible postoperative pancreas segmentation and volumetry. Automated volumetric assessment may support objective evaluation of remnant pancreatic function and provide a foundation for predictive modeling in long-term clinical management after pancreatectomy.

## 1. Introduction

The rates of detection of benign or malignant pancreatic lesions and performance of subsequent surgeries have been gradually increasing due to imaging advancements, health-screening program implementations, and population aging [1,2,3,4]. Consequently, interest in the long-term impact of pancreatic resection on the endocrine and exocrine functions of the pancreas, which significantly affect quality of life, has increased [4,5,6].

Accurate evaluation of pancreatic volume is essential because postoperative pancreatic volume is closely related to the risk of new-onset diabetes and exocrine insufficiency, which are major determinants of long-term outcomes after surgery. However, determining pancreatic volume from abdominal computed tomography (CT) scans using currently available technology is challenging, time-consuming, and impractical in clinical practice [5,6]. Manual measurements are also prone to errors and inconsistencies, limiting their reliability for routine use.

To address these limitations, a computer-aided diagnosis (CAD) system based on artificial intelligence (AI) offers a promising solution. AI-based automatic determination of pancreatic volume from abdominal CT scans could not only reduce the time and effort required in clinical practice but also improve objectivity and reproducibility in volume assessment. Moreover, automated volumetry would enable a more precise investigation of the relationship between remnant pancreatic volume and functional outcomes, thereby facilitating individualized patient management and evidence-based decision-making.

In recent years, deep learning (DL) based semantic segmentation networks have shown superior performance in medical image analysis compared with conventional methods such as intensity-based thresholding, morphology, or geometry [7,8,9]. Nevertheless, accurate pancreatic segmentation remains particularly challenging because of its structural diversity, small volume within the abdomen, and proximity to surrounding organs such as the duodenum and gallbladder [10,11]. Furthermore, standardized volumetry tools for the remnant pancreas are lacking, and most previous studies have focused on normal pancreatic anatomy or relatively small datasets [12,13]. Thus, evidence regarding volumetric changes in the remnant pancreas after surgery remains scarce.

Therefore, this study aimed to evaluate the performance of four DL-based 3D remnant pancreatic segmentation networks using a large cohort of 516 patients who underwent pancreaticoduodenectomy or distal pancreatectomy for periampullary neoplasms. Compared with prior studies, our work is distinguished by its focus on postoperative pancreatic remnants, its application of multiple advanced DL architectures, and the use of the largest postoperative CT dataset available to date. Through this approach, we demonstrate the feasibility and clinical utility of DL-based pancreatic volumetry in a postoperative setting, highlighting its potential advantages over conventional techniques.

## 2. Materials and Methods

### 2.1. Study Participants

**Figure 1:**
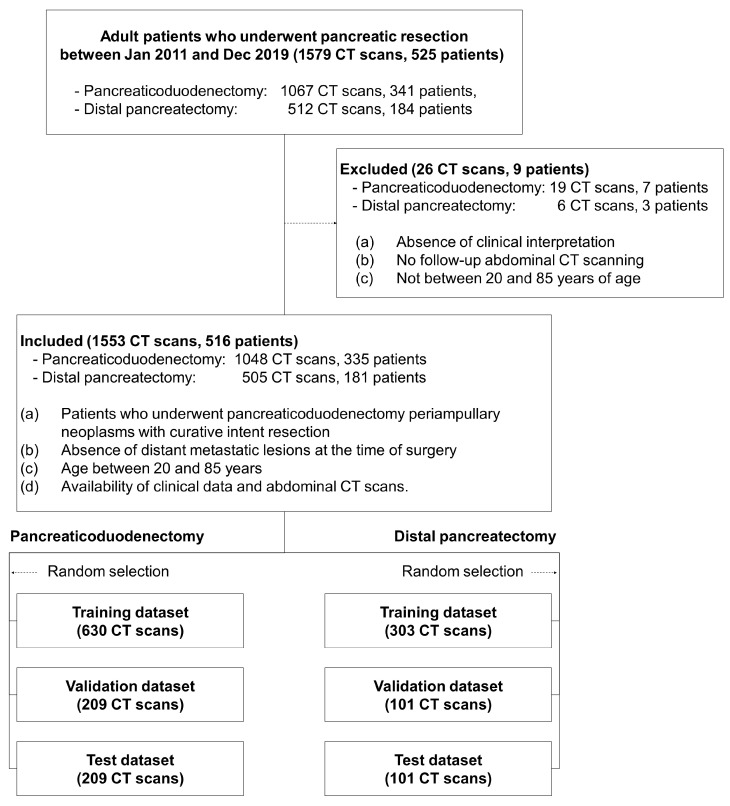
Flowchart of inclusion and exclusion criteria, highlighting the key parameters that guided patient selection.

We collected abdominal CT images from 516 patients who underwent pancreaticoduodenectomy (*n* = 335) or distal pancreatectomy (*n* = 181) for peri-ampullary neoplasms at Gil Medical Center, retrospectively reviewing clinical records between January 2011 and December 2019. This study was conducted in accordance with the Declaration of Helsinki. The Institutional Review Board of the Gil Medical Center approved this study (IRB number: GDIRB2020-121, date of approval 31 March 2020). All methods were performed in accordance with the relevant guidelines and regulations. Patient consent was waived due to the retrospective nature of the study and the use of de-identified data. The inclusion criteria for this study were: (a) patients who underwent pancreaticoduodenectomy or distal pancreatectomy for periampullary neoplasms with curative intent resection; (b) absence of distant metastatic lesions at the time of surgery; (c) age between 20 and 85 years; and (d) availability of clinical data and abdominal CT scans. The exclusion criteria were: (a) absence of clinical interpretation in the medical record, (b) lack of follow-up abdominal CT scans, and (c) age not between 20 and 85 years. Detailed information regarding inclusion and exclusion criteria is summarized in Figure 1.

### 2.2. Preprocessing

A hepatopancreatobiliary surgeon with more than 10 years of experience manually delineated the pancreas masks in 2D axial CT slices using ImageJ (ver. 1.52a; NIH, Bethesda, MD, USA) to establish the reference ground truth. In cases where postoperative changes such as fibrosis or stent-related artifacts were present, only viable pancreatic parenchyma was delineated based on enhancement patterns and anatomical landmarks, while peripancreatic fibrosis, recurrent tumor, and non-parenchymal structures were excluded. Patients with radiologic or clinical evidence of pancreatic cancer recurrence were also excluded from the analysis. Therefore, the final ground-truth annotations accurately represent the true remnant pancreatic volume after surgery, excluding postoperative changes unrelated to functional pancreatic tissue. To ensure anatomical and labeling consistency, two additional pancreatic surgeons independently reviewed and verified all delineations. The final masks were determined through a consensus-based validation process among the experts, ensuring the reliability of the manual reference labels used for model training and evaluation. The CT images obtained had varying voxel spacings, and we adjusted each image to its actual size in millimeters by considering pixel spacing and slice thickness using reflect interpolation [14]. In addition, we reconstructed the volumes of the generated masks to reflect their actual size because manual delineation was performed before volume reconstruction.

**Figure 2:**
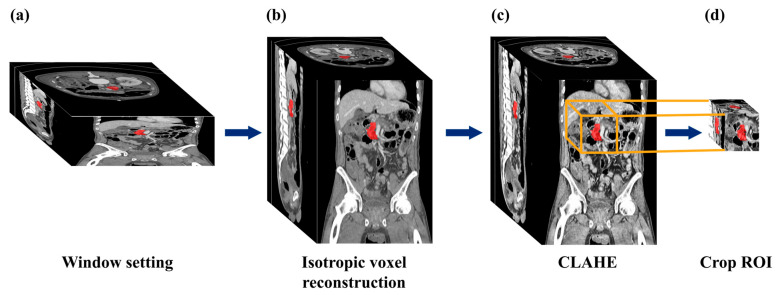
Overview of the preprocessing workflow applied to abdominal CT scans before model training. (**a**) Window setting (center = 50 HU, width = 495 HU) enhanced visualization of the pancreatic parenchyma and surrounding structures. (**b**) Isotropic voxel reconstruction resampled anisotropic slices to uniform 3D spacing. (**c**) CLAHE (Contrast-Limited Adaptive Histogram Equalization) improved local contrast and pancreas boundary visibility. (**d**) Crop ROI extracted a fixed-size region centered on the pancreas to reduce input size and focus model training. The red overlay denotes the ground-truth pancreas mask.

The pancreas is connected to neighboring organs such as the gallbladder and duodenum. Therefore, we applied contrast enhancement techniques to the input volume to improve visibility. The window center and width (50 Hounsfield Unit (HU) and 495 HU, respectively) were selected based on standard abdominal soft-tissue settings commonly used for pancreatic imaging, which optimize the visualization of the parenchyma and surrounding fat or vascular structures. These settings were applied to ensure a clear view of the abdominal region before normalization and contrast enhancement [15]. As summarized in Figure 2, preprocessing included windowing, normalization, and contrast enhancement. Intensity values between −197 HU and 297 HU were mapped to a 0–255 scale (center 50 HU). Values outside this range were clipped, creating standardized 8-bit images. To improve pancreas visibility, we applied contrast-limited adaptive histogram equalization (CLAHE). This technique involves dividing the image into multiple tiles, applying histogram equalization to each tile, and then recombining them. CLAHE parameters (clip limit = 2.0, tile size = 8 × 8) were empirically determined to enhance local contrast without amplifying noise, improving the distinction between pancreatic tissue and adjacent fat. This technique helped suppress the noise caused by excessive contrast and improve the contrast and visibility of the pancreas within the images [16,17,18,19].

Volume reconstruction and ROI cropping were essential preprocessing steps to enable valid 3D training and inference, as they ensured spatial consistency and manageable input size. In contrast, CLAHE was an optional enhancement primarily used to improve local contrast. To assess its impact, an additional experiment was conducted with and without CLAHE using the Swin U-Net, and the results are provided in Supplementary Table S1.

### 2.3. Manual Delineation

As pancreatic volume as well as its ratio to body area differed significantly among patients, using the entire abdominal area as input could negatively affect the performance of the training model. We cropped the pancreatic region, delineating its margins with guidance from the manual delineation.

For manual segmentation, we focused on CT images acquired in the early arterial or portal phases, where pancreatic tissue is well-enhanced. The process begins by locating the pancreas within the imaging data. Subsequently, we defined a region of interest (ROI) as a 128 × 128 × 128 voxel cube centered on the pancreas. A critical step involved determining the center of the pancreas in three-dimensional space to ensure its inclusion within the ROI cube. A 128 × 128 × 128 voxel cube was selected to encompass the entire pancreas after adjusting for pixel spacing and slice thickness. This yielded a single-channel 3D volume. We delineated ROIs along the outermost border of the pancreas, targeting only the pancreatic parenchyma and excluding structures such as the splenic vein, splenic artery, superior mesenteric vein, and dilated pancreatic duct that traverse the pancreas. Typically, we obtained pancreatic ROIs in approximately 30–50 transverse plane images from a single abdominal CT series.

### 2.4. Evaluation

Following these steps, we aimed to standardize the dataset, enhance pancreatic visibility, and optimize the input data to improve the performance of our model. We also used five-fold cross-validation to ensure the robustness and generalizability of the results.

For cross-validation, the dataset was divided at the patient level into five non-overlapping subsets to prevent data leakage across folds. In each iteration, three folds (60%) were used for training, one-fold (20%) for validation, and one-fold (20%) for testing. Each patient’s CT scan appeared in the test set exactly once. Final model performance was obtained by averaging the results across all five folds. Segmentation performance was assessed by pixel-wise comparison against the gold standard (threshold = 0.5). A confusion matrix (true positive, false positive, true negative, and false negative) was generated from 3D binary volume images. This process is shown in Figure 2.

### 2.5. Pancreas Segmentation with DL

We adopted 3D U-Net-based architectures for our analysis, consisting of skip connections and batch normalization, and comprising four resolution steps. Each convolution block within these architectures used a convolutional kernel size of (3 × 3 × 3), dilation rate of (1 × 1 × 1), and rectified linear units as activation functions [20,21,22,23,24,25,26].

We selected the configuration yielding the best performance for all baseline networks for the hyperparameter setting. We optimized hyperparameters using the Adam optimizer (learning rate = 0.0001) and Tversky loss as the loss function. Training was performed for up to 500 epochs with a batch size of 2, and dice_cost_0 was used as the evaluation metric. To prevent overfitting, we applied ReduceLROnPlateau (monitoring val_loss; reduction factor 0.1; patience 10; minimum learning rate (LR) = 0.000001) and Early Stopping (patience 35, based on val_loss). For the decoding steps, we opted for simple 3D up-sampling layers instead of transposed convolutional layers.

**Figure 3:**
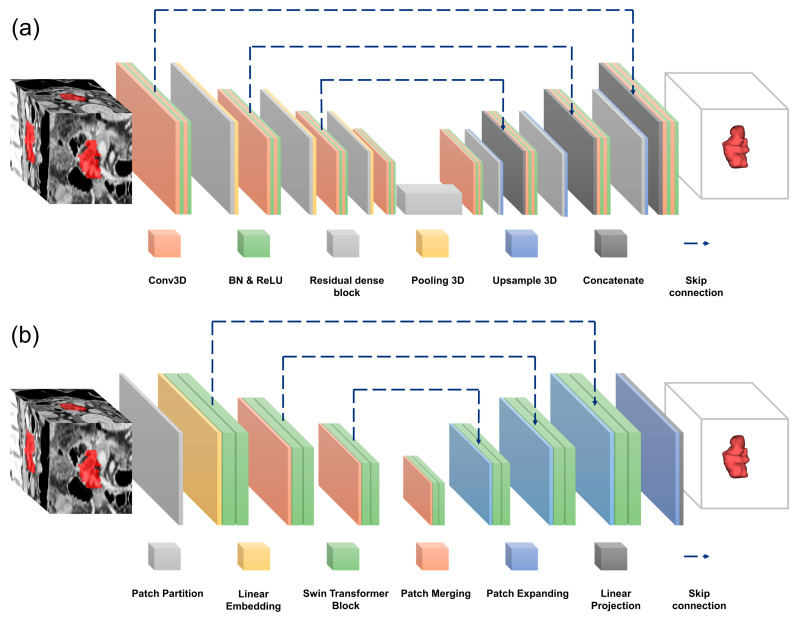
Architectures of the deep learning models used for pancreas segmentation. (**a**) Residual Dense U-Net: combines residual learning and dense connectivity to enhance gradient flow and feature reuse, enabling accurate segmentation of complex anatomical structures. (**b**) Swin U-Net: a transformer-based architecture that applies shifted-window multi-head self-attention within a hierarchical encoder–decoder framework, capturing long-range dependencies and global contextual information to improve boundary delineation of the remnant pancreas.

We replaced specific blocks with alternative modules to assess the impact of different network configurations and evaluate their effects. In our DL analysis, we utilized four convolution-based 3D U-Net architectures (basic, dense, residual, and residual dense U-Nets) with consistent widths, depths, and filter sizes while varying specific internal blocks. The architecture of the Residual Dense U-Net for pancreatic segmentation is shown in Figure 3a. To further explore recent advances in medical image segmentation, two transformer-based models (Trans U-Net and Swin U-Net) were additionally implemented and evaluated under the same preprocessing and hyperparameter settings [27,28]. Both architectures adopt a U-Net-like encoder–decoder structure but replace convolutional feature extraction in the encoder with transformer blocks, enabling the capture of long-range dependencies and global contextual information. The overall architecture of the Swin U-Net is illustrated in Figure 3b.

## 3. Results

### 3.1. Patient Characteristics

Table 1 presents the demographic characteristics of participants who underwent biliary CT after pancreaticoduodenectomy and distal pancreatectomy. Of the patients who underwent pancreaticoduodenectomy, 195 (58.2%) were male, and 140 (41.8%) were female. Their average age was 65.8 years, and their mean body mass index was 23.3 kg/m^2^. The heights (male: 167.3 ± 5.8 cm vs. female: 154.2 ± 5.7 cm) and weights (male: 64.3 ± 10.8 kg vs. female: 56.8 ± 9.2 kg) of males and females varied significantly.

Among the patients who underwent distal pancreatectomy, 88 (48.6%) were male, and 93 (51.4%) were female. Their average age was 60.2 years, and their mean body mass index was 23.2 kg/m^2^. The heights (male: 167.1 ± 5.8 cm vs. female: 155.9 ± 6.4 cm) and weights (male: 62.9 ± 9.9 kg vs. female: 58.2 ± 8.0 kg) of the males and females were significantly different. In both datasets, no significant differences were found between the ages, body mass indexes, or prevalence of hypertension and diabetes mellitus.

### 3.2. Pancreas Segmentation

**Figure 4:**
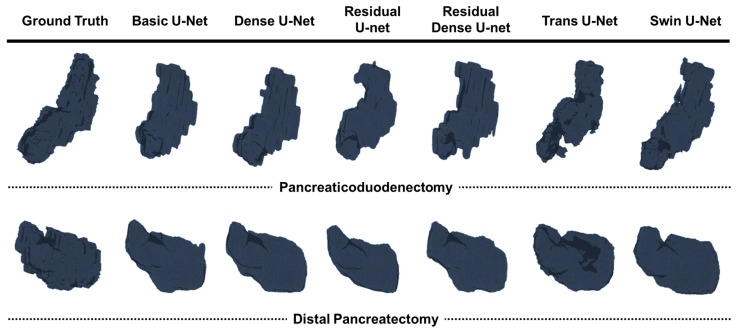
Comparison of 3D pancreas segmentation results generated by different deep learning models.

Table 2 presents the results of the five-fold cross-validation for all six 3D segmentation models, including four convolution-based U-Net variants (basic, dense, residual, and residual dense) and two transformer-based architectures (Trans U-Net and Swin U-Net), on the pancreaticoduodenectomy and distal pancreatectomy datasets, while Figure 4 illustrates representative examples of pancreatic segmentation in 2D axial slices and 3D volume renderings. Networks using residual dense blocks achieved the highest sensitivity, accuracy, and dice similarity coefficient (DSC), with very low standard deviations on both datasets [29,30,31]. Additionally, we performed paired t-tests to verify differences between the residual dense U-nets and other networks [32].

All six models demonstrated high segmentation accuracy, with Dice similarity coefficients (DSC) ranging from 0.68 to 0.81 across architectures. Among them, the Swin U-Net achieved the highest mean DSC values (0.7787 ± 0.0062 for pancreaticoduodenectomy and 0.8132 ± 0.0101 for distal pancreatectomy), followed closely by the Residual Dense U-Net (0.7655 ± 0.0052 and 0.8086 ± 0.0091, respectively).

When comparing convolution-based and transformer-based models as groups, the transformer architectures (Trans U-Net and Swin U-Net) showed slightly higher mean DSC values than the convolutional U-Net variants (*p* = 0.031 for pancreaticoduodenectomy and *p* = 0.072 for distal pancreatectomy), indicating marginal but consistent performance improvements. However, these gains were accompanied by substantially greater computational demands and longer training times, limiting their clinical practicality.

A direct comparison between the best-performing models in each group revealed that the Swin U-Net achieved marginally higher DSC values than the Residual Dense U-Net (0.7787 vs. 0.7655 for pancreaticoduodenectomy and 0.8132 vs. 0.8086 for distal pancreatectomy). Although these differences were statistically significant (*p* < 0.01), the absolute improvement was less than 2%, suggesting that while transformer-based architectures may offer slight accuracy advantages, the Residual Dense U-Net provides a more computationally efficient and clinically applicable solution with comparable segmentation accuracy.

The Residual Dense U-Net achieved mean sensitivity, specificity, precision, accuracy, and DSC of 0.7750 ± 0.0133, 0.9913 ± 0.0011, 0.8116 ± 0.0237, 0.9818 ± 0.0005, and 0.7655 ± 0.0052 for the pancreaticoduodenectomy dataset, and 0.8166 ± 0.0297, 0.9921 ± 0.0018, 0.8449 ± 0.0296, 0.9827 ± 0.0009, and 0.8086 ± 0.0091 for the distal pancreatectomy dataset, respectively.

To assess the contribution of contrast enhancement in preprocessing, we conducted an ablation of the CLAHE step using the Swin U-Net model, which achieved the highest segmentation accuracy among the tested architectures. The inclusion of CLAHE consistently improved segmentation performance, increasing the DSC from 0.733 to 0.779 for pancreaticoduodenectomy and from 0.724 to 0.813 for distal pancreatectomy. The complete results are presented in Supplementary Table S1.

We visually assessed the four semantic segmentation models in the 2D axial plane and 3D volumes based on the CT of a single patient. Three-dimensional visualization was conducted via 3D volume rendering with a VTK library [33].

### 3.3. Pancreas Volume Estimation

**Figure 5:**
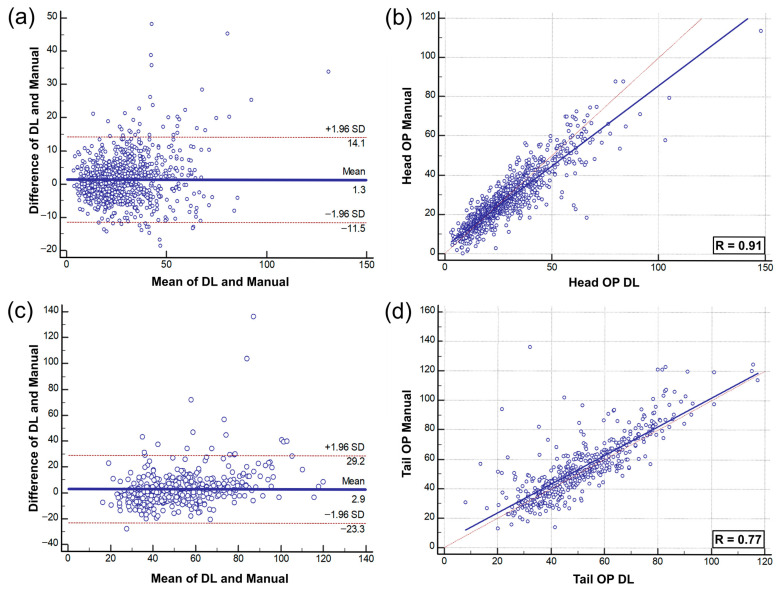
Validation of pancreatic volume estimation performance using DL and manual segmentation. (**a**,**b**) Bland–Altman and regression plots for the pancreaticoduodenectomy dataset; (**c**,**d**) Bland–Altman and regression plots for the distal pancreatectomy dataset.

We assessed the performance of the Residual Dense U-Net in estimating pancreas volume using the Bland–Altman plot and regression analysis [34,35,36] (Figure 5). In the dataset of patients who underwent pancreaticoduodenectomy, most estimation errors outside the coefficient of repeatability (±1.96 SD) were underestimated (*n* = 32); overestimations were less frequent (*n* = 11). Similarly, in the dataset of patients who underwent distal pancreatectomy, most estimation errors outside the coefficient of repeatability were underestimated (*n* = 13), with only one case of overestimation (*n* = 1). Correlation and intraclass correlation coefficient (ICC) analyses were used to evaluate the pancreatic volume. Regression analysis of the pancreaticoduodenectomy data yielded an R^2^ of 0.821 (*p* < 0.001), the correlation analysis yielded an R score of 0.910 (*p* < 0.001), and the ICC was 0.822. For the distal pancreatectomy dataset, we obtained R^2^, R, and ICC values of 0.5991 (*p* < 0.001), 0.770 (*p* < 0.001), and 0.976, respectively. Statistical analyses were performed using MedCalc Statistical Software version 23.4.0 (MedCalc Software Ltd., Ostend, Belgium).

## 4. Discussion

In recent years, advancements in medical imaging, health screening, and an aging population have led to a rise in the detection of pancreatic lesions and subsequent surgeries [1,2,3,4]. Consequently, there is growing interest in understanding the long-term effects of pancreatic resection on pancreas functions, which have a significant impact on one’s quality of life [4,5,6]. However, accurately measuring pancreatic volume from abdominal CT scans is a challenging, time-consuming, and impractical task post-pancreatectomy [5,6]. Additionally, manual measurements are prone to errors and inconsistencies. To address these issues, the study aimed to develop a CAD system based on DL technology to segment the pancreas areas and determine the volume of the remnant pancreas after pancreatectomy using abdominal CT images. In the segmentation analysis, the study used four 3D segmentation models and performed five-fold cross-validation on datasets from both surgical groups. The Residual Dense U-net trained on the pancreaticoduodenectomy dataset achieved mean sensitivity, specificity, precision, accuracy, and DSC of 0.7750, 0.9913, 0.8116, 0.9818, and 0.7655, respectively. Similarly, on the distal pancreatectomy dataset, the Residual Dense U-net obtained mean sensitivity, specificity, precision, accuracy, and DSC of 0.8166, 0.9921, 0.8449, 0.9827, and 0.8086, respectively.

Previous studies, such as those by Lim et al. and Roth et al., have demonstrated the feasibility of AI-based pancreatic segmentation in normal pancreatic anatomy or relatively limited cohorts [12,13]. However, these studies were constrained by modest dataset sizes, primarily focused on preoperative or non-surgical pancreata, and provided limited evidence for clinical applicability in postoperative settings.

Our study extends this work in several important ways. First, we analyzed the largest cohort of postoperative CT scans (*n* = 516), which allows for a more robust and generalizable evaluation of DL-based segmentation in a surgically altered pancreas. Second, we systematically compared multiple advanced DL architectures, including residual, dense, and residual dense modules, to identify the most reliable approach for remnant pancreatic segmentation. Third, and most importantly, we focused on the clinical context of post-pancreatectomy patients, in whom accurate volumetry is essential for assessing endocrine and exocrine function and guiding long-term management.

By addressing these gaps, our findings advance the field not only in terms of segmentation accuracy and reproducibility but also in clinical impact. Automated volumetry in this challenging postoperative cohort demonstrates that DL-based methods can provide consistent and objective measurements, which are difficult to achieve with manual or semi-automated techniques. Therefore, this study provides both methodological improvements and clinically meaningful insights that build upon and move beyond prior pancreas segmentation research.

Karasawa, K. et al. [9] introduced a novel atlas selection method using vessel structure around the pancreas for multi-atlas pancreas segmentation. This technique selects atlases resembling the unlabeled volume’s pancreatic features. 150 CT scans were used for the research and segmentation performances showed an average Jaccard index of 66.3% and an average Dice overlap coefficient of 78.5% for pancreas segmentation [9]. In 2021, Yan, Y. and Zhang, D. presented a 2.5D U-net with an attention mechanism for pancreas segmentation. This network combines 2D and 3D convolutional layers to capture spatial information effectively while requiring fewer computational resources than 3D models. The evaluation, conducted on 82 CT scans from the public National Institutes of Health (NIH) pancreas dataset, resulted in an average DSC of 75.33 ± 7.36, 74.04 ± 9.75, and 76.89 ± 7.44 for three views [10]. This recently published study used popular public datasets, but the limited data used in the experiment and the large variation in the performance of the trained models make it difficult to validate the performance. Holger R. et al. developed a custom 3D fully convolutional network (FCN) for automatic pancreas segmentation from abdominal CT scans. They explored two variations in the 3D FCN architecture, one with concatenation and one with summation skip connections to the decoder. The dataset of 147 CT scans from gastric cancer patients in the portal venous phase showed that the summation architecture achieved an impressive average Dice score of 89.7% with a range between 79.8% and 94.8% in testing [13]. Lim et al. achieved an average DSC of 0.850 by learning a 3D DL model on a large number of abdominal CT scans, including more than 1000 cases of normal pancreas [12]. The performance of the models presented by both teams showed excellent results with a DSC of over 80%, but data from patients before pancreatectomy was used. Overall, the study we proposed in this paper secured the validity of the experiment by collecting and using the largest number of abdominal CT images after pancreatectomy. In addition, we also used a three-dimensional DL model to accurately segment and measure the volume of the remnant pancreas after pancreatectomy, and this performance was similar to or superior to the experiment using the patient’s pancreas of normal shape and size before pancreatectomy.

It is important to note that this research utilized CT images of the pancreas from adult individuals in the Republic of Korea, specifically at the Gil Medical Center. Consequently, applying the model to anatomically distinct individuals, those with substantial variations, or minors may lead to reduced accuracy. The exclusive use of abdominal CT scans from post-pancreatic surgery patients may limit the generalizability of this model. However, this limitation could be addressed by enhancing model performance through the inclusion of abdominal CT scans from individuals of diverse ethnicities and age groups, potentially improving its generalizability. To enhance generalizability, we plan to explore domain adaptation strategies, including transfer learning, fine-tuning with small external datasets, and CT image harmonization methods. In addition, we intend to validate the model in Western populations with different anatomical and demographic characteristics. These steps will be crucial for ensuring the robustness and broader clinical applicability of our model across diverse environments. Anatomical variations in the pancreas are unlikely to significantly impact the model’s performance for calculating pancreatic volume. Measuring the pancreatic volume of patients after surgery in this study allows for the provision of objective, quantified pancreatic volume information during outpatient follow-up, alongside imaging results. Given that abdominal CT scans are typically obtained every six months following pancreatic surgery, serial pancreatic volume data can be collected using the proposed model. Utilizing this data may facilitate the development of a predictive model for pancreatic endocrine and exocrine dysfunction. While clinical assessments such as fecal elastase measurement, blood glucose levels, HbA1c, and C-peptide levels are widely used to directly evaluate exocrine and endocrine pancreatic function, these tests are limited to assessing only the current functional status of the pancreas. As pancreatic parenchyma is reduced post-surgery and due to remnant pancreatic atrophy, the risk of endocrine and exocrine dysfunction increases, making pancreatic volume data a valuable tool for prediction and interpretation [37,38]. Furthermore, the proposed model is expected to be useful for developing a predictive model for local recurrence in patients who have undergone surgery for malignant pancreatic tumors.

This study introduces a DL method aimed at accurately segmenting and measuring the volume of the remnant pancreas after pancreatectomy. The method utilizes biliary CT images from 335 patients who underwent pancreaticoduodenectomy and 181 patients who underwent distal pancreatectomy. The study focuses on DL-based pancreas segmentation using advanced 3D architectures and a large postoperative CT dataset comprising 1048 images after pancreaticoduodenectomy and 505 after distal pancreatectomy. Among the convolutional U-Net variants, the Residual Dense U-Net achieved the most accurate and stable segmentation results, owing to its combination of residual learning and dense connectivity that enhances gradient flow and feature reuse. These characteristics were particularly effective for delineating the fine pancreatic parenchyma surrounded by complex abdominal structures.

To further explore recent advances in medical image segmentation, two transformer-based architectures (Swin U-Net and Trans U-Net) were additionally evaluated alongside the convolutional U-Net family. Both transformer models demonstrated slightly higher segmentation performance than the convolution-based networks (*p* = 0.031 for pancreaticoduodenectomy and *p* = 0.072 for distal pancreatectomy), and a direct comparison between the best-performing models (Residual Dense U-Net vs. Swin U-Net) revealed a statistically significant but numerically small improvement (<2%, *p* < 0.01) in Dice similarity coefficient. These results indicate that transformer-based architectures can provide marginal accuracy gains but at the cost of markedly increased computational complexity and training time. Considering this trade-off, the Residual Dense U-Net remains the most balanced and clinically practical architecture, offering comparable accuracy with substantially greater computational efficiency.

The study also reveals a correlation between the number of trainable parameters and DL segmentation performance. Interestingly, despite the pancreaticoduodenectomy dataset being more than twice the size of the distal pancreatectomy dataset, the latter dataset proved more effective in training the model. This could be attributed to the spleen obstructing the remnant pancreas and the complexity of generating ground-truth data for pancreaticoduodenectomy cases. Additionally, the head of the pancreas was larger after distal pancreatectomy, potentially enhancing quantitative evaluation outcomes. Although the two cohorts differed in size (pancreaticoduodenectomy: 335 vs. distal pancreatectomy: 181), the performance pattern—i.e., slightly higher DSC in the distal pancreatectomy cohort despite fewer cases—suggests that sample size alone is unlikely to account for the observed differences. We interpret this discrepancy as being driven primarily by anatomical complexity and ground-truth variability rather than case counts. Although the segmentation accuracy (DSC) for the distal pancreatectomy dataset was slightly higher than that for the pancreaticoduodenectomy dataset, the correlation between predicted and manually measured pancreatic volumes (R^2^ = 0.5991 vs. 0.821) was lower. This apparent discrepancy may be explained by the markedly smaller remnant pancreatic volume after distal pancreatectomy. Because of the limited remaining tissue, even minor segmentation errors can lead to proportionally larger deviations in calculated volume, thereby reducing correlation strength in volumetric regression. In contrast, patients who underwent pancreaticoduodenectomy retained larger pancreatic remnants, resulting in smaller relative volumetric errors. These results suggest that volumetric regression accuracy is more sensitive to the absolute size of the remnant pancreas than to segmentation overlap itself. In the Bland–Altman analysis, a slight underestimation trend was observed, indicating that the predicted pancreatic volumes were marginally smaller than the manually measured ground-truth volumes. This tendency likely reflects the model’s conservative segmentation along indistinct or low-contrast pancreatic borders, particularly in postoperative regions with fibrotic change. From a clinical perspective, such a conservative bias may be preferable, as overestimation of remnant volume could misrepresent the remaining functional parenchyma. Moreover, the consistency of this bias across cases suggests that relative volumetric trends, rather than absolute values, can still be reliably interpreted in longitudinal follow-up.

Although this study primarily evaluated segmentation accuracy using metrics such as DSC, precision, and sensitivity, the clinical significance of accurate volumetry should also be emphasized. Previous studies have suggested that postoperative pancreatic volume is closely associated with the risk of new-onset diabetes mellitus, exocrine insufficiency, and nutritional status, which directly affect quality of life and long-term outcomes after pancreatectomy [37,39]. In addition, volumetric assessment may provide prognostic information regarding recurrence risk in malignant cases and support decisions on adjuvant treatment strategies. Therefore, accurate and reproducible volumetry enabled by deep learning–based segmentation could serve as a foundation for developing predictive models of postoperative pancreatic function. Future research should aim to integrate volumetric outcomes with clinical and biochemical parameters to establish risk stratification tools and individualized management strategies. By linking segmentation results to clinically meaningful endpoints, automated volumetry has the potential to move beyond technical accuracy and contribute to patient-centered care.

A limitation of this study is that the dataset was derived exclusively from Korean patients at a single institution. Anatomical and demographic variability—such as differences in body habitus, pancreatic morphology, and fat distribution—may limit the generalizability of our findings to Western populations or pediatric patients. These factors could influence the performance of segmentation networks, particularly in heterogeneous clinical settings.

Future work should therefore focus on validating our approach in multi-center and multi-ethnic cohorts, incorporating external datasets to assess reproducibility across diverse populations. In addition, domain adaptation and transfer learning techniques may help adjust the trained networks to account for anatomical variability, thereby improving robustness. Expanding to pediatric cohorts and Western institutions will be essential to confirm the broad applicability and clinical utility of automated postoperative pancreatic volumetry.

The proposed CAD program holds promise in reducing time and effort in clinical practice, serving as a valuable decision-support tool for clinicians assessing remnant pancreas post-surgery. Enhancements in CT image quality or improved differentiation of the pancreas from its neighboring organs can contribute to refining the model’s performance.

## 5. Conclusions

In this study, we developed and validated a deep learning based model for automated volumetry of the remnant pancreas after pancreatectomy using abdominal CT scans. The Residual Dense U-Net achieved a mean DSC of 0.77 and 0.81 for pancreaticoduodenectomy and distal pancreatectomy datasets, respectively, with strong correlation between predicted and manually measured pancreatic volumes (R^2^ = 0.821 and 0.599). Additional transformer-based models, including Swin U-Net, demonstrated comparable or slightly improved segmentation performance. These findings demonstrate that automated deep learning–based volumetry can provide accurate, efficient, and reproducible assessment of postoperative pancreatic volume, offering a promising tool for objective evaluation of pancreatic function in clinical practice.

## Figures and Tables

**Table 1:** Demographic and Clinical Characteristics of Patients Underwent Pancreaticoduodenectomy and Distal Pancreatectomy.

	Pancreaticoduodenectomy			Distal Pancreatectomy			*p*-Value
	Total	Male	Female	Total	Male	Female	
Number	335 (100)	195 (58.2)	140 (41.8)	181 (100)	88 (48.6)	93 (51.4)	
Age (years)	65.8 ± 10.2	64.7 ± 10.5	66.8 ± 9.7	60.2 ± 14.1	64.0 ± 11.8	56.6 ± 15.2	<0.01
Height (cm)	161.8 ± 8.6	167.3 ± 5.8	154.2 ± 5.7	161.4 ± 8.3	167.1 ± 5.8	155.9 ± 6.4	0.576
Weight (kg)	61.2 ± 10.8	64.3 ± 10.8	56.8 ± 9.2	60.5 ± 9.3	62.9 ± 9.9	58.2 ± 8.0	0.421
Body mass index (kg/m^2^)	23.3 ± 3.4	22.9 ± 3.2	23.9 ± 3.5	23.2 ± 3.3	22.5 ± 3.3	24.0 ± 3.2	0.736
Hypertension	159 (47.5)	79 (40.5)	80 (57.1)	82 (45.3)	45 (51.1)	37 (39.8)	0.621
Diabetes mellitus	101 (30.1)	60 (30.7)	41 (29.3)	55 (30.4)	32 (36.4)	23 (24.7)	0.838

This table presents the demographic and clinical characteristics of patients who underwent pancreaticoduodenectomy and distal pancreatectomy. Data are provided for the total number of patients, as well as subdivided by gender for each procedure type. The table includes information on the number of patients, their age, height, weight, body mass index, and the prevalence of hypertension and diabetes mellitus. Categorical data are presented as proportions of patients, with percentages in parentheses, and continuous data are presented as means ± standard deviation.

**Table 2:** Evaluation metrics for four pancreas segmentation models on the train, validation, and test data.

Data Set	Training Model	Sensitivity	Specificity	Precision	Accuracy	DSC	*p*-Value
Pancreaticoduodenectomy							
Train	Basic U-net	0.8934 ± 0.0235	0.9959 ± 0.0009	0.9042 ± 0.0231	0.9920 ± 0.0020	0.8936 ± 0.0251	<0.01
	Dense U-net	0.9202 ± 0.0256	0.9960 ± 0.0016	0.9098 ± 0.0360	0.9933 ± 0.0022	0.9114 ± 0.0315	
	Residual U-net	0.8676 ± 0.0184	0.9945 ± 0.0008	0.8641 ± 0.0164	0.9904 ± 0.0014	0.8590 ± 0.0192	
	Residual Dense U-net	0.8635 ± 0.0204	0.9940 ± 0.0003	0.8697 ± 0.0060	0.9886 ± 0.0012	0.8565 ± 0.0156	
	Trans U-net	0.8194 ± 0.0204	0.9975 ± 0.0004	0.8139 ± 0.0229	0.9951 ± 0.0004	0.8163 ± 0.0132	
	Swin U-net	0.8425 ± 0.0273	0.9978 ± 0.0003	0.8352 ± 0.0206	0.9957 ± 0.0005	0.8387 ± 0.0204	
Validation	Basic U-net	0.7240 ± 0.0180	0.9937 ± 0.0029	0.8157 ± 0.0208	0.9807 ± 0.0009	0.7348 ± 0.0078	<0.01
	Dense U-net	0.7418 ± 0.0206	0.9918 ± 0.0011	0.8058 ± 0.0193	0.9809 ± 0.0009	0.7419 ± 0.0079	
	Residual U-net	0.6866 ± 0.0188	0.9904 ± 0.0009	0.7707 ± 0.0197	0.9773 ± 0.0007	0.6889 ± 0.0059	
	Residual Dense U-net	0.7737 ± 0.0123	0.9916 ± 0.0010	0.8123 ± 0.0227	0.9820 ± 0.0006	0.7663 ± 0.0070	
	Trans U-net	0.7107 ± 0.0233	0.9966 ± 0.0005	0.7398 ± 0.0338	0.9928 ± 0.0003	0.7240 ± 0.0062	
	Swin U-net	0.7681 ± 0.0187	0.9973 ± 0.0001	0.7898 ± 0.0106	0.9942 ± 0.0003	0.7786 ± 0.0077	
Test	Basic U-net	0.7235 ± 0.0206	0.9924 ± 0.0013	0.8139 ± 0.0243	0.9808 ± 0.0008	0.7330 ± 0.0023	<0.01
	Dense U-net	0.7423 ± 0.0219	0.9917 ± 0.0015	0.8024 ± 0.0309	0.9809 ± 0.0007	0.7409 ± 0.0057	
	Residual U-net	0.6730 ± 0.0217	0.9909 ± 0.0011	0.7710 ± 0.0201	0.9770 ± 0.0009	0.6822 ± 0.0094	
	Residual Dense U-net	0.7750 ± 0.0133	0.9913 ± 0.0011	0.8116 ± 0.0237	0.9818 ± 0.0005	0.7655 ± 0.0052	
	Trans U-net	0.7095 ± 0.0273	0.9966 ± 0.0044	0.7385 ± 0.0211	0.9928 ± 0.0004	0.7231 ± 0.0081	
	Swin U-net	0.7693 ± 0.0215	0.9972 ± 0.0002	0.7892 ± 0.0178	0.9942 ± 0.0003	0.7787 ± 0.0062	
Distal Pancreatectomy							
Train	Basic U-net	0.9336 ± 0.0316	0.9958 ± 0.0027	0.9175 ± 0.0506	0.9932 ±0.0041	0.9213 ± 0.0462	<0.05
	Dense U-net	0.9208 ± 0.0258	0.9960 ± 0.0013	0.9175 ± 0.0258	0.9928 ± 0.0023	0.9151 ± 0.0278	
	Residual U-net	0.8992 ± 0.0082	0.9946 ± 0.0006	0.8913 ± 0.0110	0.9907 ± 0.0008	0.8900 ± 0.0099	
	Residual Dense U-net	0.8869 ± 0.0198	0.9943 ± 0.0011	0.8893 ± 0.0169	0.9896 ± 0.0019	0.8806 ± 0.0203	
	Trans U-net	0.8553 ± 0.0123	0.9966 ± 0.0007	0.8708 ± 0.0241	0.9929 ± 0.0005	0.8627 ± 0.0091	
	Swin U-net	0.8777 ± 0.0145	0.9969 ± 0.0006	0.8832 ± 0.0203	0.9938 ± 0.0007	0.8806 ± 0.0125	
Validation	Basic U-net	0.7633 ± 0.0478	0.9905 ± 0.0035	0.8092 ± 0.0532	0.9783 ± 0.0063	0.7603 ± 0.0465	<0.05
	Dense U-net	0.7673 ± 0.0480	0.9910 ± 0.0037	0.8182 ± 0.0580	0.9789 ± 0.0065	0.7676 ± 0.0496	
	Residual U-net	0.7352 ± 0.0433	0.9894 ± 0.0034	0.7865 ± 0.0514	0.9758 ± 0.0060	0.7320 ± 0.0420	
	Residual Dense U-net	0.7908 ± 0.0461	0.9905 ± 0.0040	0.8185 ± 0.0610	0.9797 ± 0.0068	0.7833 ± 0.0528	
	Trans U-net	0.6959 ± 0.0628	0.9926 ± 0.0013	0.7155 ± 0.0459	0.9849 ± 0.0027	0.7052 ± 0.0471	
	Swin U-net	0.7595 ± 0.0724	0.9938 ± 0.0014	0.7784 ± 0.0564	0.9882 ± 0.0030	0.7987 ± 0.0619	
Test	Basic U-net	0.7806 ± 0.0321	0.9924 ± 0.0013	0.8356 ± 0.0184	0.9814 ± 0.0014	0.7815 ± 0.0155	<0.05
	Dense U-net	0.7912 ± 0.0252	0.9928 ± 0.0008	0.8461 ± 0.0187	0.9821 ± 0.0010	0.7941 ± 0.0085	
	Residual U-net	0.7600 ± 0.0246	0.9910 ± 0.0009	0.8114 ± 0.0227	0.9789 ± 0.0014	0.7575 ± 0.0102	
	Residual Dense U-net	0.8166 ± 0.0297	0.9921 ± 0.0018	0.8449 ± 0.0296	0.9827 ± 0.0009	0.8086 ± 0.0091	
	Trans U-net	0.7393 ± 0.0353	0.9931 ± 0.0007	0.7321 ± 0.0231	0.9860 ± 0.0008	0.7250 ± 0.0165	
	Swin U-net	0.7839 ± 0.0184	0.9950 ± 0.0004	0.8060 ± 0.0153	0.9896 ± 0.0006	0.8132 ± 0.0101	

This table compares the performance of four different U-net models (Basic U-net, Dense U-net, Residual U-net, and Residual Dense U-net) in segmenting CT images for patients who underwent pancreaticoduodenectomy and distal pancreatectomy. The metrics evaluated include sensitivity, specificity, precision, accuracy, and DSC. Each metric is presented with means ± standard deviations for both types of surgical procedures.

## Data Availability

The data presented in this study are available on request from the corresponding author due to patient privacy protection and ethical restrictions under IRB regulations.

## Supplementary Materials

The following supporting information can be downloaded at: https://www.mdpi.com/article/10.3390/diagnostics15222834/s1, Table S1. Comparison of segmentation performance with and without CLAHE preprocessing using the Swin U-Net model for pancreaticoduodenectomy and distal pancreatectomy datasets.

## Abbreviations

The following abbreviations are used in this manuscript:

AI	artificial intelligence
CAD	computer-aided diagnosis
CLAHE	contrast-limited adaptive histogram equalization
CT	computed tomography
DL	deep learning
DSC	dice similarity coefficient
FCN	fully convolutional network
HU	Hounsfield units
ROI	region of interest
