# Supplementary material for: Deep Learning Model for Volume Measurement of the Remnant Pancreas After Pancreaticoduodenectomy and Distal Pancreatectomy
Source: Diagnostics (Basel). 2025 Nov 8;15(22):2834. doi: 10.3390/diagnostics15222834 (PMC12651614; doi:10.3390/diagnostics15222834)
Supplement: Supplementary file 1 [file diagnostics-15-02834-s001.zip › diagnostics-3874575-supplementary.pdf]

diagnostics

MDPI

Table S1. Comparison of segmentation performance with and without CLAHE preprocessing using the Swin U-Net model for pancreaticoduodenectomy and distal pancreatectomy datasets.

Data Set	Training Model	CLAHE	Sensitivity	Specificity	Precision	Accuracy	DSC	p-value
Pancreaticoduodenectomy (Head operation)								
Train	Swin U-net	X	0.8378 ± 0.0114	0.9998 ± 0.0001	0.7930 ± 0.0120	0.9998 ± 0.0001	0.8147 ± 0.0084	< 0.01
	Swin U-net	O	0.8425 ± 0.0273	0.9978 ± 0.0003	0.8352 ± 0.0206	0.9957 ± 0.0005	0.8387 ± 0.0204	
Validation	Swin U-net	X	0.7255 ± 0.0303	0.9998 ± 0.0001	0.7409 ± 0.0074	0.9996 ± 0.0001	0.7327 ± 0.0133	< 0.01
	Swin U-net	O	0.7681 ± 0.0187	0.9973 ± 0.0001	0.7898 ± 0.0106	0.9942 ± 0.0003	0.7786 ± 0.0077	
Test	Swin U-net	X	0.7322 ± 0.0147	0.9998 ± 0.7343	0.7343 ± 0.0150	0.9996 ± 0.0001	0.7330 ± 0.0096	< 0.01
	Swin U-net	O	0.7693 ± 0.0215	0.9972 ± 0.0002	0.7892 ± 0.0178	0.9942 ± 0.0003	0.7787 ± 0.0062	
Distal Pancreatectomy (Tail operation)								
Train	Swin U-net	X	0.7744 ± 0.0201	0.9998 ± 0.0001	0.8324 ± 0.0152	0.9996 ± 0.0001	0.8062 ± 0.0141	< 0.01
	Swin U-net	O	0.8777 ± 0.0145	0.9969 ± 0.0006	0.8832 ± 0.0203	0.9938 ± 0.0007	0.8806 ± 0.0125	
Validation	Swin U-net	X	0.6698 ± 0.0393	0.9997 ± 0.0001	0.7501 ± 0.0270	0.9994 ± 0.0001	0.7027 ± 0.0301	< 0.01
	Swin U-net	O	0.7595 ± 0.0724	0.9938 ± 0.0014	0.7784 ± 0.0564	0.9882 ± 0.0030	0.7987 ± 0.0619	
Test	Swin U-net	X	0.6562 ± 0.0651	0.9997 ± 0.0001	0.7495 ± 0.0189	0.9993 ± 0.0001	0.7235 ± 0.0272	< 0.01
	Swin U-net	O	0.7839 ± 0.0184	0.9950 ± 0.0004	0.8060 ± 0.0153	0.9896 ± 0.0006	0.8132 ± 0.0101	
